# H3K27ac mediated SS18/BAFs relocation regulates JUN induced pluripotent-somatic transition

**DOI:** 10.1186/s13578-022-00827-1

**Published:** 2022-06-16

**Authors:** Runxia Lin, Ziwei Zhai, Junqi Kuang, Chuman Wu, Yuxiang Yao, Ruona Shi, Jiangping He, Shuyang Xu, Pengli Li, Yixin Fan, Wei Li, Zichao Wu, Xiaoxi Li, Jin Ming, Jing Guo, Bo Wang, Dongwei Li, Shangtao Cao, Xiaofei Zhang, Yi Li, Duanqing Pei, Jing Liu

**Affiliations:** 1 CAS Key Laboratory of Regenerative Biology, Guangzhou Institutes of Biomedicine and Health, Chinese Academy and Sciences, Guangzhou, 510530 China; 2 Guangdong Provincial Key Laboratory of Stem Cell and Regenerative Medicine, Guangzhou Institutes of Biomedicine and Health, Chinese Academy of Sciences, Guangzhou, China; 3 Bioland Laboratory (Guangzhou Regenerative Medicine and Health Guangdong Laboratory), Guangzhou, 510005 China; 4 Laboratory of Cell Fate Control, School of Life Sciences, Westlake University, Hangzhou, 310024 China; 5 University of Chinese Academy of Science, Beijing, 100049 China; 6 GMU-GIBH Joint School of Life Sciences, Guangzhou Medical University, Guangzhou, China; 7 School of Physical Science and Technology, Lanzhou University, Lanzhou, China; 8 CAS Key Laboratory of Regenerative Biology, Guangdong Provincial Key Laboratory of Pathogenesis, Targeted Prevention and Treatment of Heart Disease, Guangzhou Institutes of Biomedicine and Health, Chinese Academy of Sciences, Guangzhou, China; 9 Guangdong Provincial Key Laboratory of Pathogenesis, Targeted Prevention and Treatment of Heart Disease, Guangdong Provincial People’s Hospital, Guangdong Academy of Medical Sciences, Guangzhou, 510100 Guangdong China

**Keywords:** Embryonic stem cells, Differentiation, Early development, PST, Chromatin organization, SS18/BAFs, H3K27ac

## Abstract

**Background:**

The exit from pluripotency or pluripotent-somatic transition (PST) landmarks an event of early mammalian embryonic development, representing a model for cell fate transition.

**Results:**

In this study, using a robust JUN-induced PST within 8 h as a model, we investigate the chromatin accessibility dynamics (CAD) as well as the behaviors of corresponding chromatin remodeling complex SS18/BAFs, to probe the key events at the early stage of PST. Here, we report that, JUN triggers the open of 34661 chromatin sites within 4 h, accomplished with the activation of somatic genes, such as *Anxa1*, *Fosl1*. ChIP-seq data reveal a rapid relocation of SS18/BAFs from pluripotent loci to AP-1 associated ones. Consistently, the knockdown of *Brg1*, core component of BAF complexes, leads to failure in chromatin opening but not closing, resulting in delay for JUN induced PST. Notably, the direct interaction between SS18/BAFs and JUN-centric protein complexes is undetectable by IP-MS. Instead, we show that H3K27ac deposited by cJUN dependent process regulates SS18/BAFs complex to AP1-containing loci and facilitate chromatin opening and gene activation.

**Conclusions:**

These results reveal a rapid transfer of chromatin remodeling complexes BAF from pluripotent to somatic loci during PST, revealing a simple mechanistic aspect of cell fate control.

**Supplementary Information:**

The online version contains supplementary material available at 10.1186/s13578-022-00827-1.

## Background

Cell fate transition is a multi-level, high-precision, multivariate-regulated intricate biological event, which extensively occur in multi-physiological processes, such as organism development [1, 2], immunity [3, 4], stress [5], metabolism [6], and tumorigenesis [7, 8]. The exit from pluripotency or pluripotent-somatic transition (PST) is a prospective cell fate transition model [9], representing a key stage for early embryonic development, however, remains largely unknown in mechanisms. Basically, previous models for PST research mostly focus on the changing of the ESC culture medium, such as removal of LIF [9–11], or addition of FGF2 [12] etc., with considerable heterogenous and instability, limited the underline mechanism research.

Previously, we reported that JUN/AP-1, acting as guarder of somatic cell fate, is incompatible with OCT4, which safeguard the pluripotent cell fate in mouse embryonic stem cells or mESCs [13]. The induction of JUN leads to a dramatic morphological change as well as repression of pluripotent genes and activation of somatic genes within 48 h in ESCs [13]. Based on these findings, we construct a robust and fast PST system, JUN^TetON^ ESC [13]. Compared to other PST models (2–3 days) [9–11, 14], JUN-induced PST takes within only 8 h (less than one cell cycle), more than 6 h JUN induction will lead an irreversible differentiation of mESCs [15], thus offering us a good opportunity to capture the key events of PST occurred in the early stage. Recently, we reported that SS18/BAFs play a critical role in JUN-induced PST [15]. BAFs, also known as mammalian switch/sucrose nonfermentable(mSWI/SNF), initially found in S. *cerevisiae*, contain an enzyme subunit BRG1 or BRM serving the ATP-dependent chromatin remodeling function, as well as other regulatory subunits, like SS18 [16]. BAF complexes have three subtypes: canonical BAF (cBAF), non-canonical BAF (ncBAF) and polybromo-associated BAF (PBAF). They share the ATPase BRG1 but differ in specific subunits [17]. Our recent work shows that SS18 contained BAFs, i.e., cBAF and ncBAF, but not PBAF, facilitate JUN induced PST [15]. BAFs play crucial roles in many biological processes, including but not limited to development [18], immunity [3, 19, 20] and cancer [16]. However, the underlying mechanism on how BAFs regulate JUN-induced PST is still unknown.

Given the remarkable impacts of SS18/BAFs on chromatin architecture, we investigate the chromatin accessibility dynamics as well as the behaviors of SS18/BAFs by using ATAC-seq, ChIP-seq and IP-MS techniques to detect the detail information on how SS18/BAFs respond to JUN induction signaling to mediate the quick PST in 8 h. Here, we report that, instead of direct protein–protein interaction with JUN centric protein complex, SS18/BAFs undergoes a large-scale relocation from pluripotent loci to AP-1 binding loci by recognizing the H3K27ac created by JUN overexpression, leading to a rapid PST.

## Results

### Chromatin accessibility dynamics during JUN-induced PST

Taking advantages of the incompatibility between JUN and pluripotency, we constructed a robust PST system, JUN^TetON^ ESC, in which 90% mESCs colonies (~ 90%) will exit from pluripotency within 8 h [15], providing us an ideal platform to investigate the underline mechanisms for cell-fate transition with time scale in hours. To evaluate the non-specific effect for Doxycycline (Dox) concentration, we test the response of WT ESC by 2 μg/ml Dox treatment. No significant change was observed in either the expression of pluripotent/AP-1 related genes (Additional file 1: Fig. S1A) or the morphology of ESCs (Additional file 1: Fig. S1B). In addition, knockdown JUN by shRNA in JUN^TetON^ ESC, can partially rescue Dox induced PST (Additional file 1: Fig. S1C). Those data indicate that the non-specific effect for 2 μg/ml Dox treatment in JUN-induced PST system is extremely low.

**Fig.1:**
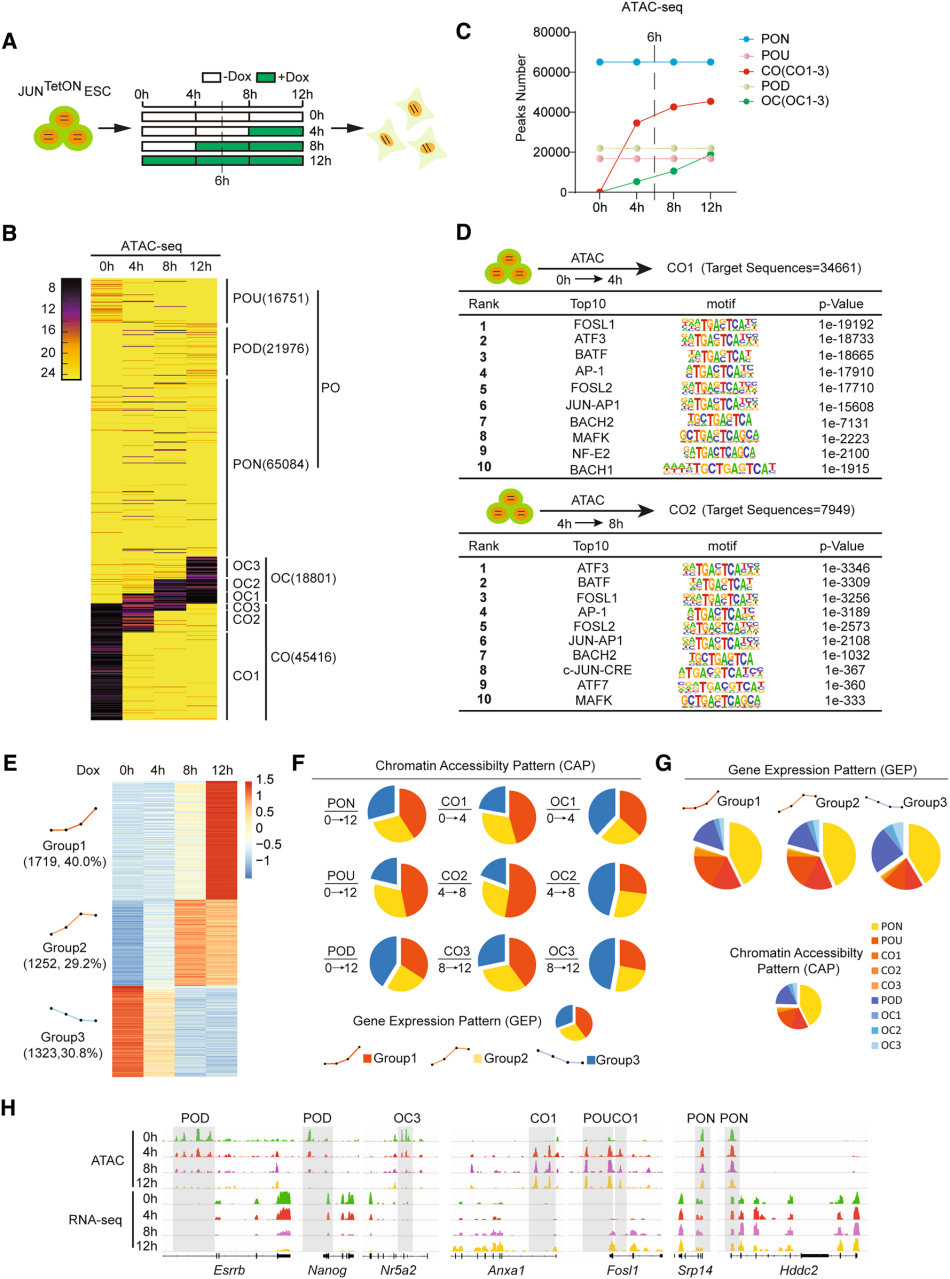
The chromatin accessibility dynamics during PST. **A** Schematic for JUN induced pluripotent to somatic transition (PST) and time course sample collection. ESCs containing JUN^TetON^ and switched into medium plus/minus doxycycline(Dox) and then harvested for ATAC-seq at the time points indicated in the upper row. Time points: 0 h, 4 h, 8 h, 12 h. **B** Heatmap showing the chromatin accessibility dynamic changes during PST. Loci of chromatin were arranged into groups depending upon the day of PST they changed from closed to open (CO) or open to closed (OC) or when they were permanently open (PO). PO was subdivided into those loci that declined (but remained open) as PO-down(POD), those that increased as PO-up(POU), and those that were unchanged (PON). CO and OC were subdivided CO1-3, OC1-3 respectively. **C** The number of peaks defined in each of the OC/CO and PON/POU/POD categories. **D** Motif analysis for CO1 and CO2 during JUN based PST. Table lists for the top10 most enrichment motifs. Peaks number = 34,661, 7949, respectively. **E** Heatmap of RNA-seq data during JUN based PST. Three groups have been divided, gruop1 and group2 indicate gene upregulated, and group3 indicates gene downregulated. **F** Correlation between ATAC-seq and RNA-seq. Each group of ATAC-seq binding sites are mapped to the TSS–/ + 10 kb, these gene are overlap to RNA-seq. Then we calculate the percentage of three group RNA-seq in each ATAC group which got by overlap ATAC-seq and RNA-seq, indicated by pie chart. **G** Calculating the percentage of every ATAC group in each RNA-seq group which got by overlap ATAC-seq and RNA-seq, indicated by pie chart. **H** Selected genomic views of the ATAC-seq data were shown for the indicated PON, POU, POD, CO, OC groups. Loci of indicated groups are marked with gray boxes. The RNA-seq expression values for the respective genes are shown below. RNA-seq expression units are in TPM (Transcripts Per Million reads), the following is the same

To figure out the chromatin architecture dynamics during JUN-induced PST, we collected JUN^TetON^ mESCs samples with Dox treatment for 0, 4, 8 and 12 h (Fig. 1A) for ATAC sequencing (ATAC-seq). The calling peaks, as shown previously [21], was divided into three basic categories: PO, permanently open during PST, which was further divided into PO-up (POU),PO-down (POD) and PO-no change(PON) subgroups according to the trends of chromatin accessibility dynamics; OC, open at 0 h but closed during PST, which was further divided into OC1(0–4 h), OC2(4–8 h) and OC3(8–12 h) subgroups according to different time window; CO, closed at 0 h but opened during PST, which was further divided CO1(0–4 h), CO2(4–8 h) and CO3(8–12 h) subgroups according to different time window (Fig. 1B). Based on the above catalogs, we show by heatmap (Fig. 1B) and line chart (Fig. 1C) that the chromatin state change dramatically by JUN induction within 12 h, e.g., 18,801 OC peaks, 45,416 CO peaks, 16,751 PO-up peaks and 21,976 PO-down peaks. Strikingly, the curve of CO1 peaks shows the largest increments (34,661) among all the subgroups (Fig. 1C). As 6 h exposure to Dox treatment leads to an irreversible pluripotency exit during JUN induced PST [15], we further investigate the chromatin accessibility dynamics (CAD) features for those subgroups in detail. Motif discovery for each subgroup indicates a gradual chromatin closing for loci occupied by pluripotent factors, e.g., ESRRB, TCFCP2L1, NR5A2, TCF3/4, KLF4/5, POU5F1, SOX2, OCT4-SOX2-TCF, while prominent chromatin opening for loci occupied by AP-1 factors, e.g., JUND, FOSL1/2, ATF1/2/3/4/7, BACH1/2, MAFA/MAFK (Additional file 2: Fig. S2A, B), demonstrating the closing of pluripotent chromatin and the opening of somatic chromatin in JUN-induced PST, consistent with the binary logic we proposed for cell fate transition in reprogramming [21]. We then show the top10 motifs enriched for each CAD subgroups. Significantly, motifs for AP-1 family factors are the dominant ones in all the CO1, CO2 and CO3 peaks, while motifs for SOXs/OCTs were obvious in all the OC1, OC2 and OC3 peaks (Fig. 1D and Additional file 2: Fig. S2B). Notably, motif for YY1 is present in OC1 and OC3, but not in OC2, indicating the difference for chromatin closing among those three stages (Additional file 2: Fig. S2B).

By combining RNA-seq and ATAC-seq data, we further investigate the correlation between gene expression pattern (GEP) and chromatin accessibility pattern (CAP) during PST. To achieve this, the differentially expressed genes are divided into three groups: Group 1, gradually upregulated,1719; Group 2, immediately upregulated,1250; Group 3, down regulated,1323 (Fig. 1E and Additional file 2: Fig. S2C). We extracted all the genes located in each CAP subgroups and made a mapping for the genes shared between each CAP subgroup and GEP subgroup (Fig. 1F, G). The mapping ratios are calculated to assess the correlation between GEP and CAP. Through simple statistical analysis, we show that, PON relative genes have similar ratios to GEP pattern; POU/CO1/CO2 relative genes have the most significant correlation with gene upregulation; POD/OC3/OC2 relative genes have the most significant correlation with gene down-regulation (Fig. 1F). In addition, genes in Group1/2 have higher mapping ratio to POU/CO1/2/3; Genes in Group3 have higher mapping ratio to POD/OC1/2/3 (Fig. 1G).

We further investigate the relationship between CAP and GEP in temporal dimension by analyzing specific genes in the time course RNA-seq and ATAC-seq data. We find a significant delay for the emergence of RNA peaks (8 h) to ATAC peaks (4 h) in the loci of somatic genes such as *Anxa1*, *Fosl1* (Fig. 1H). These findings suggest a model in which chromatin open facilitating gene activation at the early stage of PST. In addition, the behaviors of ATAC peaks and RNA peaks in the loci of pluripotent relative genes such as *Esrrb*, *Nanog*, and *Nr5a2* are quite synchronous, both disappearing at 12 h (Fig. 1H). These data indicate that JUN mediated chromatin opening initiates the transition of PST.

### The relocation of SS18/BAFs during JUN-triggered PST.

**Fig. 2:**
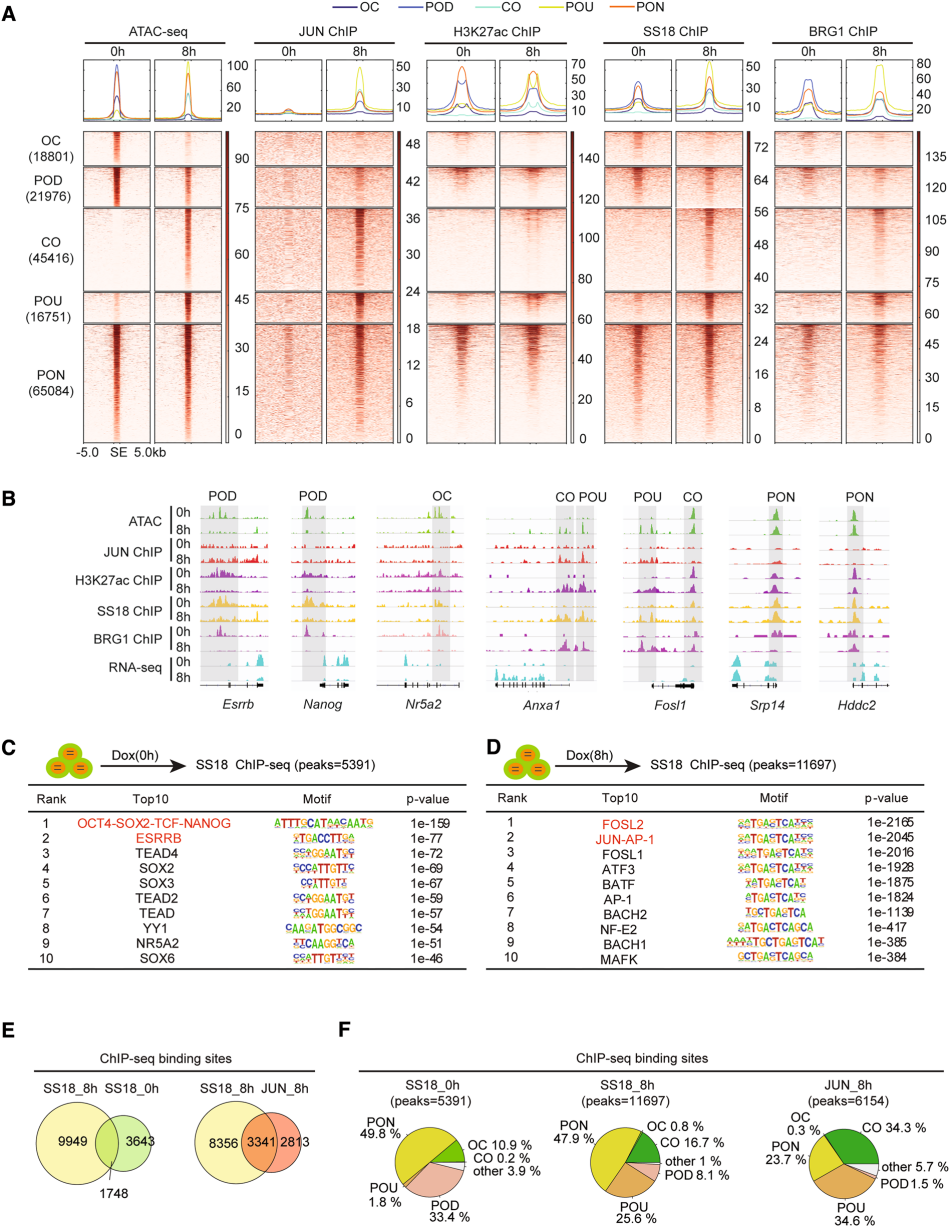
SS18/BAFs relocate from Pluripotent loci to somatic ones. **A** The combination analysis for the ATAC-seq, JUN ChIP-seq, SS18 ChIP-seq and BRG1 ChIP-seq, H3K27ac ChIP-seq data. All the ATAC-seq occupancy sites ± 5 kb of 0hs and 8 h were divided into five categories which were same with Fig. 1B. Number indicates peaks counts of each category. SE stands for TSS and TES, the following is the same. **B** Representative OC, CO, PON, POU, POD genomic views of multiple omics dataset including ATAC-seq, ChIP-seq and RNA-seq under BAFs/SS18 and JUN regulations during the process of JUN based PST and are marked in grey boxes. **C** ChIP-seq analysis for SS18 at 0 h during JUN based PST. Table list for the top10 most enrichment motifs. Peaks number = 5391. **D** ChIP-seq analysis for SS18 at 8 h during JUN based PST. Table list for the top10 most enrichment motif. Peaks number = 11,697. **E** Overlapped peaks for JUN and SS18 at 0 h and 8 h as indicated during JUN based PST indicated in venn plot. **F** Venn plot show changes in chromatin accessibility during the exit of pluripotency at the 0 and 8 h SS18 binding sites and at 8 h JUN binding sites

Recently, using CRISPR/CAS9 genome-wide screening technology, we identified SS18/BAF complexes as critical epigenetic coagent for JUN-triggered PST [15]. To further explore the underline mechanisms, we performed ChIP-seq to detect the occupancy of JUN, SS18/BAFs in chromatin as well as the modification of H3K27ac during PST at 0 h and 8 h, respectively. The resulting omics data were combined to ATAC-seq data for further analysis (Fig. 2A). Generally, heatmaps for these omics data reveal a genome-wide consistency in pattern among chromatin accessibility state, JUN binding and H3K27ac modification (Fig. 2A, left three panel), consistent with the function of JUN protein in chromatin opening. Attractively, ChIP-seq signaling for SS18 and BRG1 show a significant switch from OC/POD peaks at 0 h to CO/POU peaks at 8 h (Fig. 2A, right two panel), indicating a dramatic relocation for SS18/BAFs during PST. Particularly, in loci relative to pluripotent genes, such as *Esrrb*, *Nanog* and *Nr5a2*, the loss of SS18/BRG1 binding is accompanied with chromatin closing, loss of H3K27ac as well as mRNA expression (Fig. 2B). In contrast, for loci related to somatic genes, such as *Anxa1* and *Fosl1*, the emergency of SS18/BRG1 binding signaling is accompanied with chromatin opening, gain of H3K27ac, as well as mRNA expression (Fig. 2B). We then performed motif discovery for SS18/BRG1 binding sites at 0 h and 8 h, respectively. Top10 enriched motifs indicates an obvious translocation of SS18 from loci regulated by pluripotent factors, such as OCT4-SOX2-TCF-NANOG, ESRRB, NR5A2, SOX2 at 0 h to AP-1 family factors, such as FOSL2, JUN, FOSL1, ATF3, BATF, BACH1/2 at 8 h (Fig. 2C, D), the later one was largely overlapped to the motifs enriched in JUN binding loci at 8 h (Additional file 3: Fig. S3A). Quantitatively, SS18 undergoes a relocation from 3643 chromatin loci at 0 h to 9949 new chromatin loci at 8 h, remains only 1748 loci unchanged (Fig. 2E, left panel). Notably, JUN shares substantial co-occupancy with SS18 at 8 h (Fig. 2E, right panel), indicating a possible protein–protein interaction between JUN and SS18. We further investigate the chromatin accessibility dynamics (CAD) for loci occupied by SS18 (0 h, 8 h) and JUN(8 h), respectively, and showed by pie chart that, among loci occupied by SS18 at 0 h (5391), PON, 49.8%, indicating a constant open in chromatin state for those loci to 8 h; POD + OC,33.4% + 10.9%, indicating a majority close for those loci to 8 h (Fig. 2F, left panel). While for loci occupied by SS18 at 8 h (11,697), PON, 47.6%, POU + CO, 25.6% + 16.7%, represented a majority open for those loci to 8 h(Fig. 2F, middle panel). Those data indicate SS18 has a strong correlation with chromatin open. In addition, for loci occupied by JUN at 8 h (6154), PON (23.7%) + CO(34.3%) + POU(34.6%), 92.6% in sum (Fig. 2F, right panel), also suggests a significant correlation for JUN in chromatin open. Taken together, those data suggested a widely relocation for the binding of SS18/BAFs in chromatin at the early stage during JUN induced PST.

### SS18/BAFs regulate PST through chromatin opening

**Fig. 3:**
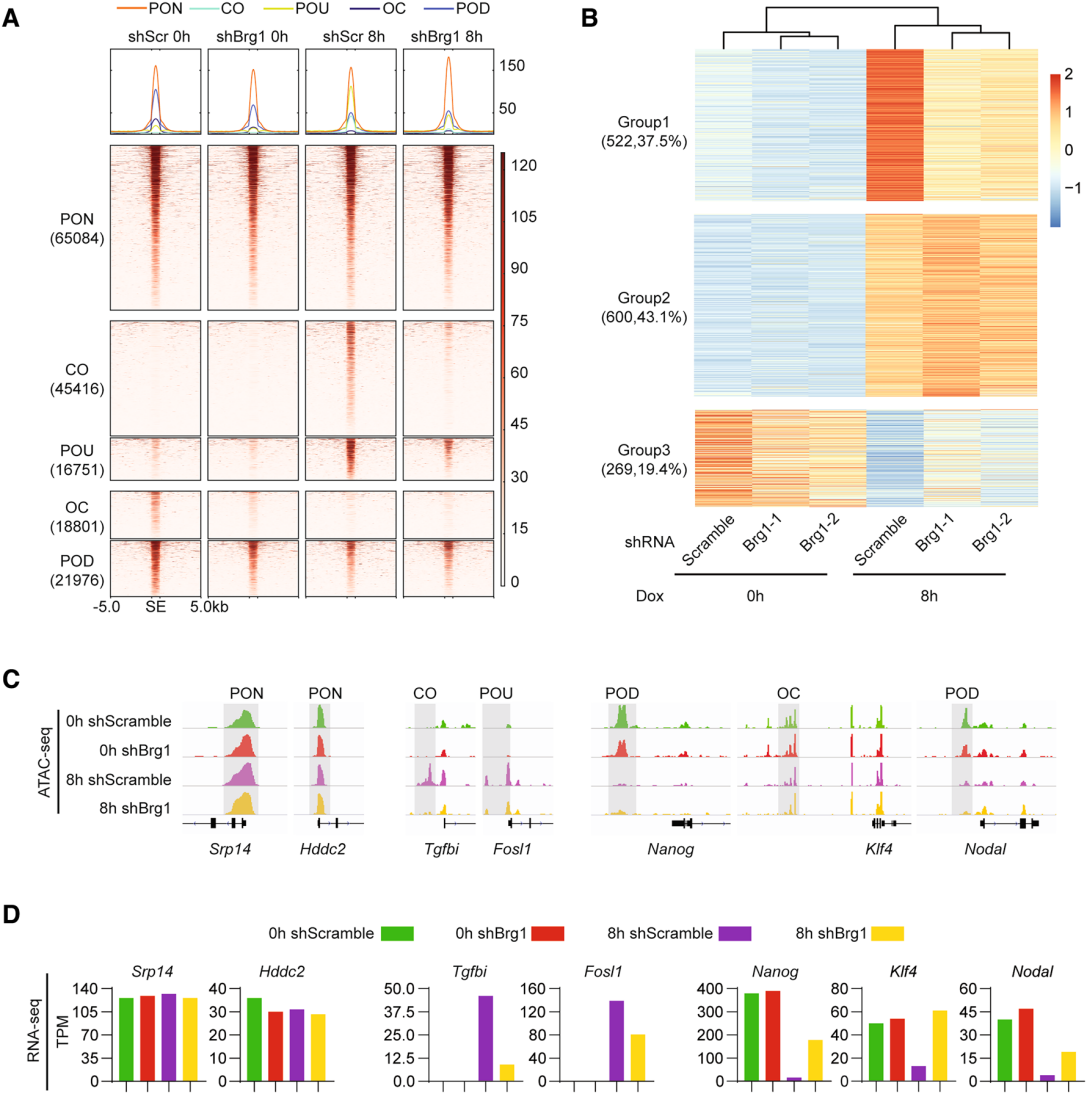
BAFs deficiency will derail JUN-triggered PST. **A** Heatmap of ATAC-seq during Jun plus/minus Brg1 knock down based PST. The five categories were same with Fig. 1B. **B** Heatmap of Brg1 shRNA RNA-seq during JUN based PST, time point 0 h and 8 h. Differential expression gene(DEG) divided three group according shScramble gene expression change between 0 and 8 h. Fold change 1.5. **C** Selected genomic views of the ATAC-seq data are shown for the indicated PON, POU, POD, CO, OC groups. Loci of indicated groups are marked with gray boxes. **D** The RNA-seq expression values for the respective genes in Figure 3C selected genomic views were shown in the bar plot

Given the correlation among SS18/BAFs relocation, JUN binding pattern, chromatin accessibility dynamics, as well as the function of BAFs in chromatin remodeling, we hypothesize that SS18/BAFs may regulate PST by promoting the accessibility for JUN binding to its targets. To this end, we knocked down *Brg1*, the core ATPase of BAFs by shRNA and then performed ATAC-seq to detect the change of chromatin accessibility during PST. Heatmaps show a dramatic deficiency in CO/POU sites upon the absence of BRG1, while has little impact on the ones in OC/POD/PON groups at 8 h (Fig. 3A), suggesting the major function of BRG1 is chromatin opening. We further compared the chromatin accessibility state between shScr and shBrg1 at 8 h (Additional file 4: Fig. S4A). *Brg1* knockdown leads 55,098 ATAC signaling down (ATD), 17,345 ATAC signaling up (ATU), as well as 60,086 ATAC signaling permanent (ATP) at 8 h. Motif discovery for ATD shows extremely significant for AP-1 family factors, such as FOSL1, JUNB, FOSL2 (Additional file 4: Fig. S4B), suggested the dominant control of these loci by AP1 family factors. Intriguingly, CTCF binding sites are dominant in ATP and ATU groups (Additional file 4: Fig. S4A, B). Meanwhile, we analysis the impact on transcriptome by *Brg1* knockdown at 8 h during PST, and showed by heatmap that, 522 genes are failure to activation (Fig. 3B, up panel, and Additional file 4: Fig. S4C), 600 genes have no significant impact (Fig. 3B, middle panel, and Additional file 4: Fig. S4D), and 269 genes are failure to silence (Fig. 3B, down panel, and Additional file 4: Fig. S4E). We further examined the gene expression pattern for specific loci within different CAD groups upon *Brg1* knockdown, and show that *Srp14* and *Hddc2*, belongs to PON in CAD during PST has no change upon *Brg1* knockdown in both chromatin accessibility state and gene expression (Fig. 3C, D, left panel); *Tgfbi* and *Fosl1*, JUN targeting genes, belongs to POU/CO1 in CAD during PST, present a failure in chromatin open, accompanied with recession in gene activation, upon *Brg1* knockdown at 8 h (Fig. 3C, D, middle panel); *Nanog*, *Klf4*, *Nodal*, pluripotent genes, belongs to OC/POD group in CAD during PST, present a failure in chromatin close, accompanied with delay in gene repression (Fig. 3C, D, right panel).These data suggest that SS18/BAFs are critical for chromatin open in AP-1 binding loci at the early stage during JUN induced PST.

### SS18/BAFs are not associated with JUN centric protein complexes

**Fig. 4:**
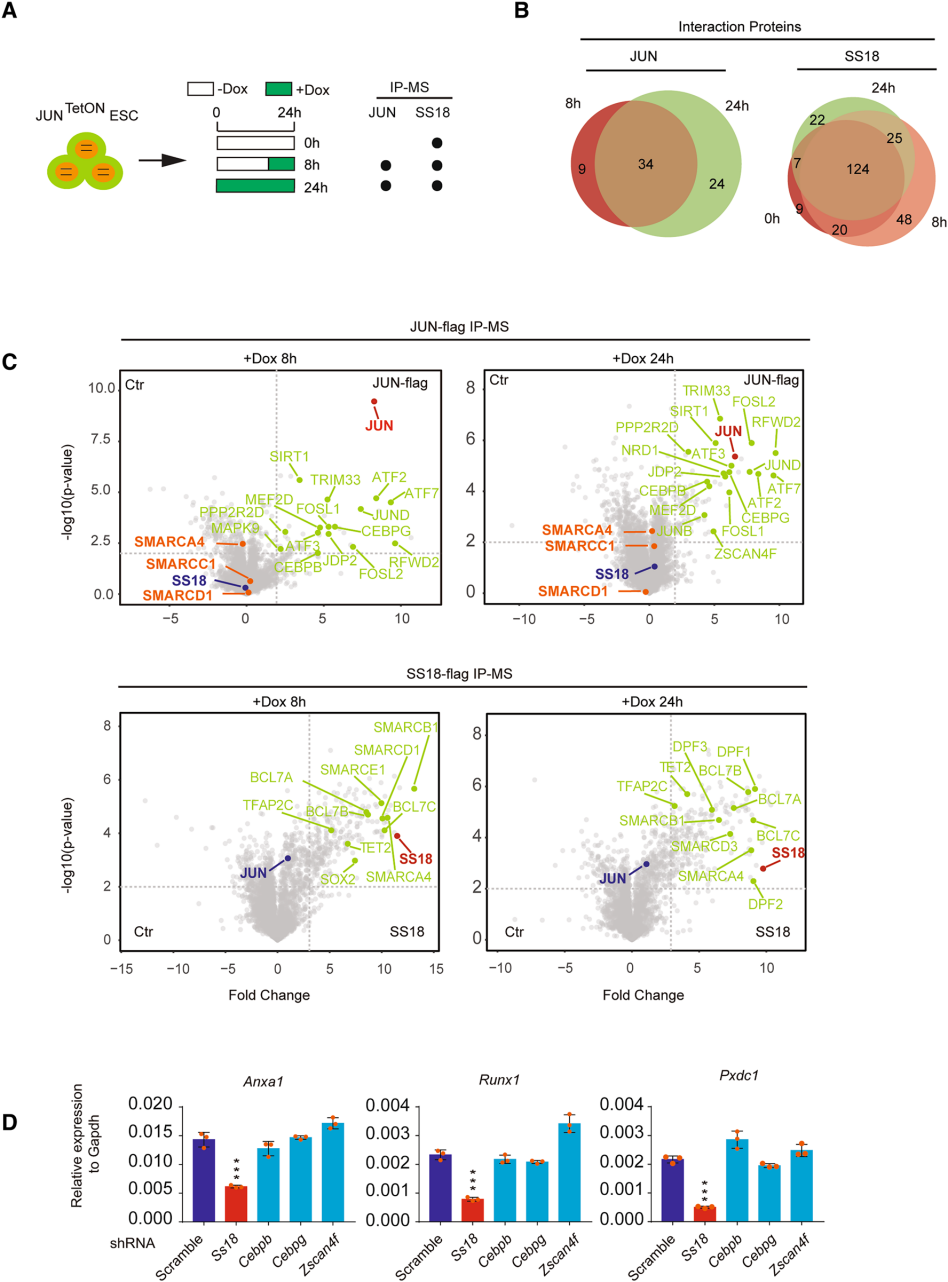
Protein–protein interaction cannot account for BAFs relocation. **A** Schematic illustration of Mass Spectrometry after Immunoprecipitation experiments. Three time points 0 h, 8 h and 24 h were utilized to analyze the proteome of JUN and SS18. **B** Venn plots showing the overlaps of proteins that interact with JUN and SS18 at different time points during PST, respectively. **C** The scatterplots above showed the pairwise comparison between 8 h or 24 h and 0 h during PST by Flag antibody in JUN-Flag^TetON^mESCs with label-free quantification. The proteins of JUN, SS18 and the representative ones interacted with JUN were marked in red, blue, yellow and green respectively. The scatterplots below showed the pairwise comparison between SS18 antibody and IgG isotype control at 8 h and 24 h during PST in Jun-Flag^TetON^ mESCs with label-free quantification. The proteins of SS18, JUN and the representative ones interacted with SS18 were marked in red, blue and green respectively, P-value = 0.01 and fold change = 2 were used as threshold. Every point represented a single protein. IP-MS experiments were performed in triplicate and a two-sample t-test was applied. **D** The expression of representative downstream genes of *Jun* were detected by real-time quantification PCR at 8 h during PST upon the knockdown of the shared three proteins. Data are mean ± s.d., two tailed, unpaired t-test; n = 3 independent experiments, ***P < 0.001

The massive and rapid relocation of SS18/BAFs from pluripotent loci to AP-1 binding loci raised a possibility that JUN centric protein complexes may mediate SS18/BAFs relocation by direct protein–protein interaction. To test this hypothesis, we performed IP-MS for JUN or SS18 to detect any possible interaction between JUN-centric protein complex to SS18/BAFs. As the probable protein–protein interaction would be more stable at later stage of PST, we collect JUN^TetON^ ESC samples with DOX treatment for 0, 8 h and 24 h to detect the putative interaction (Fig. 4A). Venn plots showed the dynamics in composition for JUN centric or SS18-centric protein complexes by dox treatment with different time duration (Fig. 4B). Specifically, JUN centric protein complexes shared 34 members at 8 h and 24 h, while SS18 centric protein complexes shared 124 members at 0, 8 and 24 h (Fig. 4B), indicating a high stability for SS18/BAF complexes. We then focused on the interaction between SS18 and JUN. Scatterplots showed that neither JUN nor SS18 could pull down each other in IP-MS data (Fig. 4C). To find any possible protein–protein interaction(s) links JUN and SS18, we built SS18 centric and JUN centric network based on IP-MS data (Additional file 5: Fig. S5A, B). Here, we show that cBAF/ncBAF complexes, the super elongation complex (SEC) and nucleosome remodeling and deacetylase (NuRD) are shared in SS18 centric complex in 0, 8 and 24 h (Additional file 5: Fig. S5A), while AP-1 family members are shared in JUN centric network at 8 and 24 h (Additional file 5: Fig. S5A). Although no obvious interaction between AP-1 family members and subunits within BAF complexes was observed, three proteins, CEBPB, CEBPG and ZSAN4F are shared in both SS18 and JUN centric complexes at 8 h (Additional file 5: Fig. S5A, B), which may link JUN and SS18 during PST. To test this hypothesis, we knocked down those genes by shRNA (Additional file 5: Fig. S5C), and then tested the expression of JUN downstream genes, such as *Anxa1*, *Runx1* or *Pxdc1* by RT-PCR, no inhibitory effect was detected, as SS18 shRNA did during PST (Fig. 4D), suggesting function irrelevant for these three proteins to PST. Interestingly, when analysis time course IP-MS data for SS18 by heatmap (Additional file 5: Fig. S5D), we found NANOG and ESRRB, two key transcriptional factors, undergone a gradually separation from SS18/BAFs at 8 h and 24 h, respectively, suggesting a decoupling of SS18/BAFs for pluripotency maintain during PST (Additional file 5: Fig. S5D).

Together, these data suggest that the relocation of SS18/BAFs to target loci is unrelated to direct interaction between JUN centric complexes and SS18/BAFs.

### SS18/BAFs recognize JUN induced H3K27ac signaling for relocation

Given no evidence support the relocation of SS18/BAFs mediated by direct interaction with JUN centric protein complexes, we turned to other mechanisms. BAF complex have been reported mainly co-localized with active histone modification such as H3K27ac, H3K4me1/3, etc. [22]. In addition, Polycomb complex dependent histone modification, such as H3K27me3, H2AK119ub have been shown incompatible with BAF complex [18]. To this end, we performed ChIP-seq for H3K4me1, H3K4me3, H3K9ac, H3K27me3 and H2AK119ub, together with the existing H3K27ac data to investigate the relationship between those histone modifications and SS18/BAFs during JUN induced PST. As shown by heatmap, the occupancy of SS18 in genomic have significant overlap with active histone modification, but barely no overlap with H3K27me3 and H2AK119ub (Additional file 6: Fig. S6A). Venn diagrams show the exact amount of co-occupancy sites between SS18 and those histone modifications at 0 h and 8 h, respectively (Additional file 6: Fig. S6B and S6C). Among all the active histone modification, H3K27ac have the largest overlap with SS18 in binding sites during JUN induced PST(Additional file 6: Fig. S6A–C), indicating a high correlation between H3K27ac and SS18, thus raised the hypothesis that the relocation of SS18/BAFs may regulated by recognizing of H3K27ac signaling during PST.

**Fig. 5:**
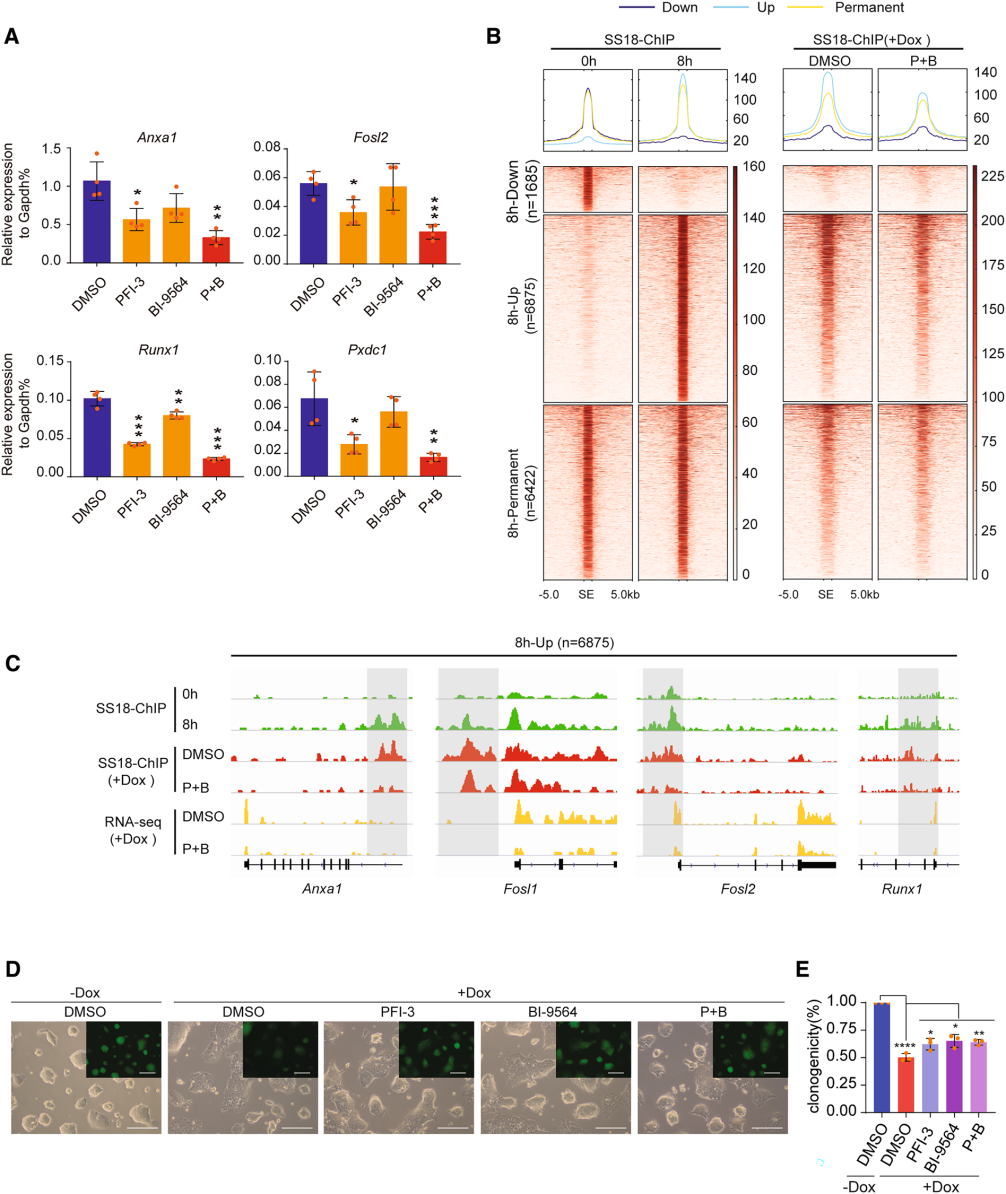
H3K27ac mediates SS18/BAFs relocation and PST. **A** The expression of representative downstream genes of *Jun* were detected by real-time qPCR at 6 h during PST with 30 μM Brg1 bromodomain inhibitor, PFI-3 (P) and 30 μM Brd7/Brd9 bromodomain inhibitor, BI-9564 (B) treatment, and combined P + B. Data are mean ± s.d., two tailed, unpaired t-test; n = 4 independent experiments, *P < 0.05, **P < 0.01, ***P < 0.001. **B** ChIP-seq heatmap showing the SS18 dynamic changes and SS18 ChIP-seq from P + B and DMSO during PST. Loci of chromatin were arranged into three groups depending upon the fold change of strength of SS18 binding peaks between 0 and 8 h. Fold change 2. Down stands for 8 h compare to 0 h < 2, number of peaks = 1685, up stands for 8 h compare to 0 h > 2, number of peaks = 6875, the others peaks stand permanent, number of peaks = 6422. **C** Selected genomic views of ChIP data and RNA-seq data are shown for the indicated Down, Up, Permanent groups. Loci of indicated groups are marked with gray boxes. **D** Representative images for 48 h in 2i/LIF medium after c-Jun induction with different treatment, DMSO(Dox 0 h) DMSO(Dox 6 h), PFI-3(30 μM, Dox 6 h), BI-9564(30 μM, Dox 6 h), P + B(15 μM + 15 μM, Dox 6 h) respectively. Three biological replicates. **E** Clongenicity of OCT4-GFP positive colony recovered for 48 h in 2i/LIF medium after c-Jun induction with different treatment, DMSO(Dox 0 h) DMSO(Dox 6 h), PFI-3(30 μM, Dox 6 h), BI-9564(30 μM, Dox 6 h), P + B(15 μM + 15 μM, Dox 6 h) respectively. Data are mean ± s.d., two-sided, unpaired t test; n = 3 independent experiments. **P < 0.01, ****P < 0.0001

It was report that H3K27ac could be catalyzed by recruitment of CBP/P300 at JUN binding sites and recognized by proteins that contain bromodomain in BAF complex [23–26]. To this end, we test the above hypothesis by using two compounds, PFI-3, a BRG1 bromodomain (H3K27ac reader) inhibitor, and BI-9564, a BRD7/BRD9 bromodomain inhibitor, to block the BAF dependent H3K27ac recognition [25, 26]. Here, we show by RT-PCR that single or combined application of these two inhibitors, especially the later, could significantly block the activation of JUN target genes, such as *Anxa1*, *Fosl2*, *Runx1* and *Pxdc1* (Fig. 5A). We further performed RNA-seq to investigate the impact of the inhibitors on transcriptome at 8 h during PST. In detail,179 JUN upregulated genes were disturbed by the two inhibitors (Additional file 7: Fig. S7A and S7B). The disturbed genes are involved in the processes of cell-substrate adhesion, positive regulation of cell adhesion (Additional file 7: Fig. S7C), consistent with the morphologic change during JUN induced PST. We further performed ChIP-seq experiments to detect the location of SS18 upon the treatment of the two inhibitors at 8 h during PST, and show by heatmap that the binding of SS18 to new loci was reduced significantly (Fig. 5B). RT-PCR analysis further confirmed the repression of JUN target genes, such as *Anxa1, Fosl2*, *Fosl1* and *Runx1* by these two inhibitors (Fig. 5C). Furthermore, the JUN induced PST, measuring by cellular morphology and clonogenicity, was delay by the application of the two inhibitors (Fig. 5D, E). Taken together, these data suggest that H3K27ac links JUN to SS18/BAFs relocation in JUN induced PST.

## Discussion

**Fig. 6:**
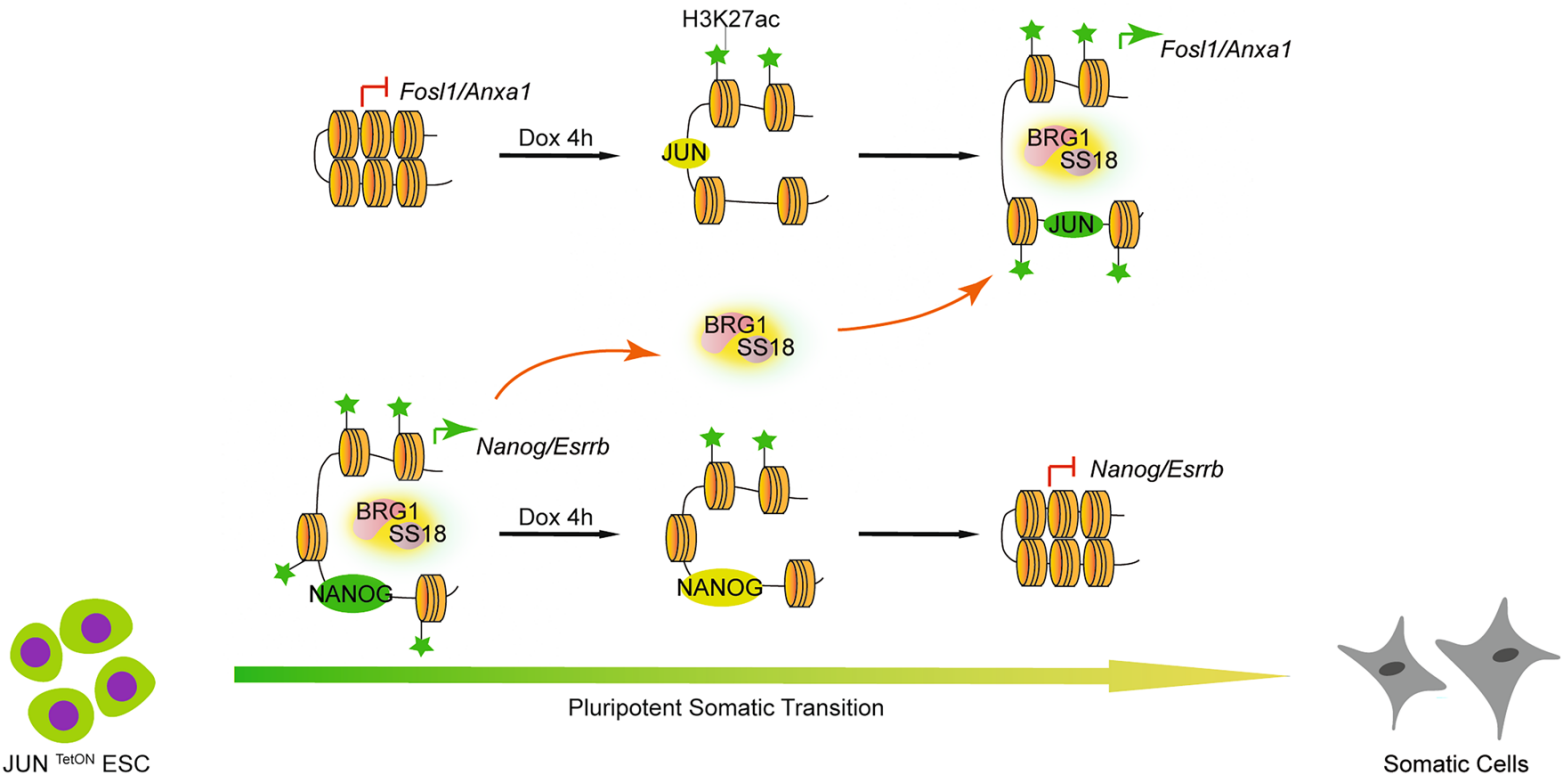
Working model. **A** Working model for JUN induced PST. In embryonic stem cells, opened chromatin marked by H3K27ac are occupied by SS18/BAF and pluripotent transcriptional factors. The induction of cJUN for 4 h can trigger deposition of H3K27ac in cJUN/AP-1 binding sites, which are further recognized by SS18/BAF, leading the opening of the target chromatin and activation of somatic genes. The green asterisk represents H3K27ac

Previously, we proposed that JUN/AP-1 could act as the guardian for somatic cell fate [13, 21], similarly to OCT4 as a guardian for pluripotent cell fate. In parallel to somatic-to-pluripotent transition(SPT) or somatic reprogramming driven by defined factors [27, 28], we established the opposite process, i.e., pluripotent-to-somatic transition (PST) in less than 24 h by taking advantage of JUN induction in mESCs, and further identified the critical corresponding epigenetic machinery SS18/BAFs [15]. In this study, we further investigated the underline mechanisms, especially the key events happened at the early stage of PST. By carefully study the chromatin accessibility dynamics, behaviors of SS18/BAFs as well as the histone modification within the first 8 h, we found that the induction of JUN trigged amount of chromatin opening only within 4 h, accomplish with rapid SS18/BAFs relocation from pluripotent loci to those newly open loci, as well as the activation of somatic genes and deactivation of pluripotent genes. Importantly, we found that the relocation of SS18/BAFs is not mediated by direct interaction among transcriptional factors and epigenetic machine but linked by the histone modification H3K27ac. (Fig. 6A).

During JUN induced PST, the induction of JUN leads a rapid H3K27ac. Previous studies reported that JUN and FOS can form heterodimer to recruit BAFs directly to participate in enhancer selection in mouse embryonic fibroblasts (MEFs) upon serum stimulate [29, 30]. In addition, TFs such as CBP/P300, CEBPB [31], SMAD1 [32] and ATF2 [33] have been reported can interact with JUN and involved in acetyltransferase activity in different cell context. Thus, the identification of the specific enzyme that catalyzing H3K27 acetylation during PST can offer new insights into the directed differentiation of stem cells.

In this report, we found that the recognition of H3K27ac plays a crucial role in SS18/BAFs relocation. However, other epigenetic modifications, such as H3K4me1/3 seems also involved in JUN induced PST (Additional file 6), and could also recognized by proteins in SS18/BAF complex [34, 35]. The function of different epigenetic modifications as well as their crosstalk during PST deserve further study.

## Conclusions

Taken together, our results reveal a concise orchestration among transcription factor, histone modification and epigenetic machine during cell fate transition. JUN-induced PST model could serve as a powerful tool for further mechanisms research in cell fate regulation.

## Methods

### Cell culture

All the animal experiments were performed with the approval and according to the guidelines of the animal care and use committee of the Guangzhou Institutes of Biomedicine and Health. HEK293T cells are cultured in DMEM supplemented with 10% FBS, GlutaMAX and NEAA. Mouse ESCs are maintained under feeder-free condition with mESC-2i/LIF medium containing DMEM, 15% FBS, NEAA, GlutaMAX,PD0325901 (Targetmol T6189), Chir99021 (Targetmol T2310), LIF (MilliporeESGE107). HEK293T was obtained from ATCC (CRL-1126). mESCs were derived in-house by crossing Oct-GFP trans genetic allele carrying male mice (male CBA/ cAJ x female C57bl/6 J) and female 129/Sv mice. All the cell lines have been confirmed as mycoplasma free with Lonza LT07-318. LIF/2i are pulsed in the culture medium continuously, the final concentration of Dox used in this report is 2 μg/ml.

### Lentivirus‑mediated shRNA transfection

ShRNA lentivirus were constructed into pLKO.1 vector according to operation manual. Sequence of indicated shRNA are provided in Additional file 8: Table S1. shRNA target sequences. Lentiviruses were produced using HEK293T cells, collect virus supernatant after 48 h transfection.

### qRT–PCR and RNA-seq

Total RNAs were prepared with TRIzol. For quantitative PCR, cDNAs were synthesized with ReverTra Ace (Toyobo) and oligo-dT (Takara), and then analysed by qPCR with ChamQ SYBR qPCR Master Mix (Vazyme). The qRT-PCR primers used in this study are provided in Additional file 9: Table S2 RT-qPCR primers. VAHTS mRNA-seq V3 Library Prep Kit for Illumina (NR611, Vazyme) was used for library constructions and sequencing done with NextSeq500 Mid output 150 cycles (FC-404–2001, Illumina) for RNA-seq. RNA-seq data was analyzed by DEseq2, GO.db and Mfuzz. Processed RNA-seq in this study are provided in Additional file 11: Table S4 RNA-seq.

### ChIP-Seq

The ChIP-seq was constructed with NovoNGS CUT&Tag 2.0 HighSensitivity Kit for Illumina (novoprotein N259-YH01) according to manufacturer’s instructions. ChIP-seq data are mapped to the 10 mm mouse genome assembly using bowtie2, version 2.4.1. Information about antibody are provided in Additional file 13: Table S6 Antibody.

### ATAC-seq

ATAC-seq was performed as previously described (Buenrostro et al., 2013; Buenrostro et al., 2015a). In brief, a total of 50,000 cells were washed once with 50 mL of cold PBS and resuspended in 50 mL lysis buffer (10 mM Tris–HCl pH 7.4, 10 mM NaCl, 3 mM MgCl2, 0.2% (v/v) IGEPAL CA-630). The suspension of nuclei was then centrifuged for 10 min at 500 g at 4 °C, followed by the addition of 50 mL transposition reaction mix (25 mL TD buffer, 2.5 mL Tn5 transposase and 22.5 mL nuclease-free H2O) of Nextera DNA library Preparation Kit (96 samples) (FC-121–1031, Illumina). Samples were then PCR amplified and incubated at 37 °C for 30 min. DNA was isolated using a MinElute Kit (QIAGEN). ATAC-seq libraries were first subjected to 5 cycles of pre-amplification. To determine the suitable number of cycles required for the second round of PCR the library was assessed by quantitative PCR as described (Buenrostro et al. 2015a), and the library was then PCR amplified for the appropriate number of cycles. Libraries were purified with a Qiaquick PCR (QIAGEN) column. Library concentration was measured using a KAPA Library Quantification kit (KK4824) according to the manufacturer’s instructions. Library integrity was checked by gel electrophoresis. Finally, the ATAC library was sequenced on a NextSeq 500 using a NextSeq 500 High Output Kit v2 (150 cycles) (FC-404-2002, Illumina) according to the manufacturer’s instructions.

### SS18 endogenous immunoprecipitation and on-bead digestion

Whole cellextracts of mES cells with cJun overexpression were prepared using lysis buffer (50 mM Tris pH 8.0, 150 mM NaCl, 10% Glycerol, 0.5% NP40) with fresh added 1 × Complete Protease inhibitors (Sigma, 1187358001). Cells were incubated for 2 h at 4 °C on a rotation wheel. Soluble cell lysates were collected after maximum speed centrifugation at 4 °C for 15 min. 1 mg of cell lysates were incubated with either SS18 antibody or matched IgG overnight at 4 °C on a rotation wheel. Combined Protein A/G magnetic beads (Bio-rad, 1614833) were added for another 1.5 h. Beads were then washed three times with wash cell lysis buffer and one time with PBS. After completely removal of PBS, immunoprecipitated proteins were digested using on-bead digestion protocol as described before 17. Briefly, beads were incubated with 100 mL of elution buffer (2 M urea, 10 mM DTT and 100 mM Tris pH 8.5) for 20 min. Afterwards, iodoacetamide (Sigma, I1149) was added to a final concentration of 50 mM for a 10 min in the dark, following with 250 ng of trypsin (Promega, V5280) partially digestion for 2 h. After incubation, the supernatant was collected in a separate tube. The beads were then incubated with 100 mL of elution buffer for another 5 min, and the supernatant was collected in the same tube. All these steps were performed at RT in a thermo shaker at 500 g. Combined elutes were digested with 100 ng of trypsin overnight at RT. Finally, tryptic peptides were acidified to pH < 2 by adding 10 mL of 10% TFA (Sigma, 1002641000) and desalted using C18 Stage tips (Sigma, 66,883-U) prior to MS analyses. Each experiment was performed in technical triplicate.

### Mass spectrometry analysis

Tryptic peptides were separated by AcclaimTM PepMapTM 100 C18 column (Thermo, 164,941) using a 140 min of total data collection (100 min of 2–22%, 20 min 22–28% and 12 min of 28–36% gradient of B buffer (80% acetonitrile and 0.1% formic acid in H2O) for peptide separation, following with two steps washes: 2 min of 36–100% and 6 min of 100% B buffer) with an Easy-nLC 1200 connected online to a Fusion Lumos mass spectrometer (Thermo). Scans were collected in data-dependent top-speed mode with dynamic exclusion at 90 s. Raw data were analyzed using MaxQuant version 1.6.0.1 search against Mouse Fasta database, with label free quantification and match between runs functions enabled. The output protein group was analyzed and visualized using DEP package as described before. Processed IP-mass date are provided in Additional file 10: Table S3 IP-MS.

### Statistical information

Data are presented as mean ± s.d. as indicated in the figure legends. Unpaired two-tailed student t test, The P value was calculated with the Prism 6 software. A P < 0.05 was considered as statistically, *P < 0.05, **P < 0.01, ***P < 0.001. No statistical method was used to predetermine sample size. The experiments were not randomized. The investigators were not blinded to allocation during experiment and outcome assessment.

### Data and software availability

The RNA-Seq, ChIP-seq data have been deposited in the Gene Expression Omnibus database under the accession code GSE135451 and GSE186175and in the National Center for Biotechnology Information under the accession number PRJNA843614.

The mass spectrometry proteomics data have been deposited to the ProteomeXchange Consortium (http://proteomecentral.proteomexchange.org) via the iProX partner repository [36] with the dataset identifier PXD029277.

## Supplementary Information

Additional file 1. System specificity of JUN^TetON^ ESCs.

Additional file 2. ATAC motif analysis and RNA-seq Gene ontology.

Additional file 3. JUN motif analysis.

Additional file 4. BRG1 is required for JUN to open chromatin.

Additional file 5. SS18/BAFs and JUN form independent complexes.

Additional file 6. H3K27ac co-occupancy with SS18 during JUN induced PST.

Additional file 7. The BAFs’ bromodomain inhibitors impede PST.

Additional file 8. shRNA target sequences used in this study.

Additional file 9. RT-qPCR primers used in this study.

Additional file 10. IP-MS data in this study.

Additional file 11. RNA-seq data in this study.

Additional file 12. ATAC-seq TSS binding sites data in this study.

Additional file 13. Antibody used in this study.

Additional file 14. Descriptions text (Please replace this with a new additional file 14 in the attachments file which we made a minor correction).

## Data Availability

The authors declare that all data supporting the findings of this study are available within the article and its supplementary information files or from the corresponding author upon reasonable request. Source data are provided with this paper.

## Abbreviations

PST: Pluripotent-somatic transition
CAD: Chromatin accessibility dynamics
CO: Chromatin open
PO: Permanent open
OC: Open to close
POU: PO-up
POD: PO-down
PON: PO-no change
mSWI/SNF: Mammalian switch/sucrose nonfermentable
cBAF: Canonical BAF
ncBAF: Non-canonical BAF
PBAF: Polybromo-associated BAF
ATAC-seq: ATAC sequencing
SEC: Super elongation complex
NuRD: Nucleosome remodeling and deacetylase
PHD: Plant homeodomain
