# Supplementary figures and images for: H3K27ac mediated SS18/BAFs relocation regulates JUN induced pluripotent-somatic transition
Source: Cell Biosci. 2022 Jun 16;12:89. doi: 10.1186/s13578-022-00827-1 (PMC9204951; doi:10.1186/s13578-022-00827-1)

Fig. S1

A

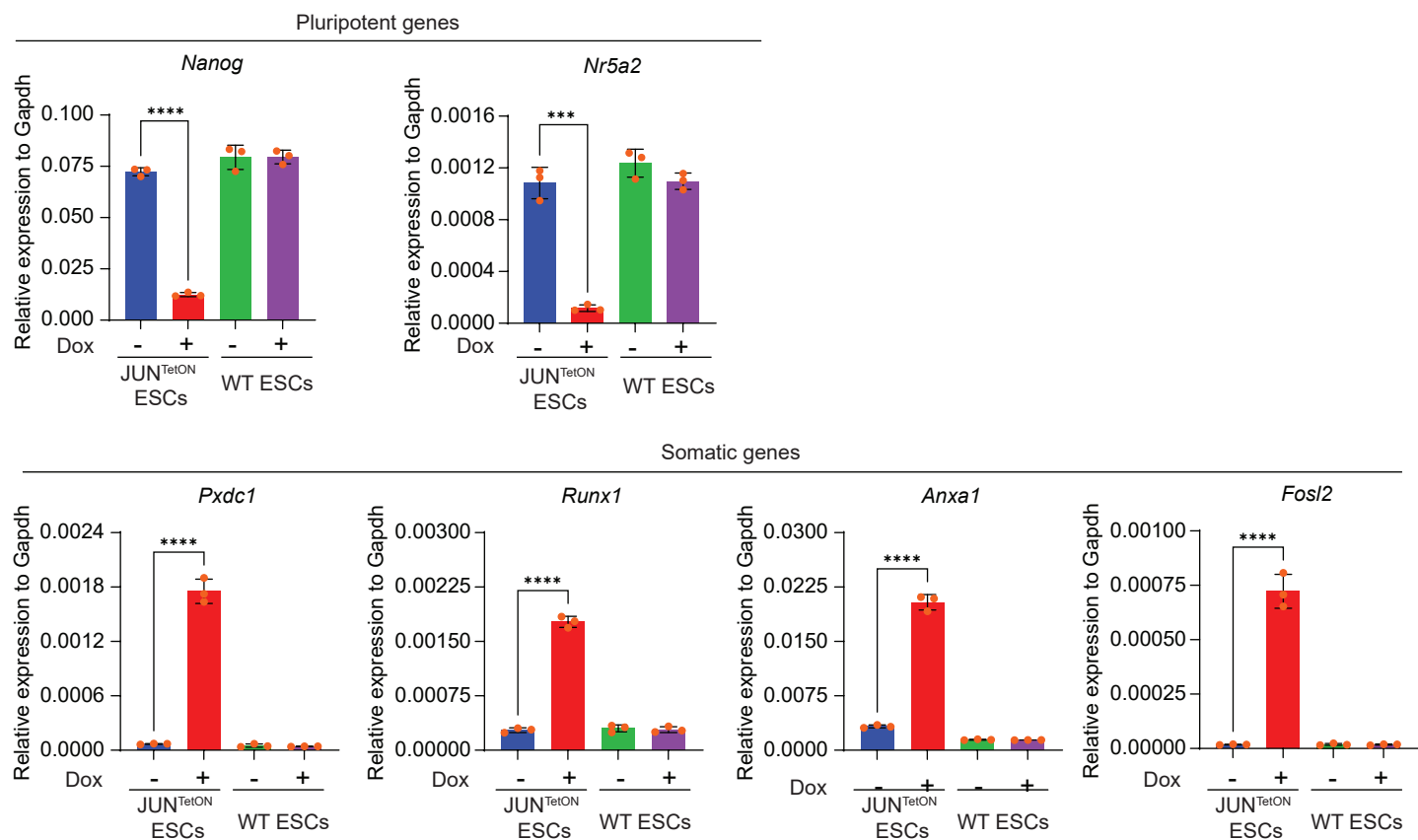

B

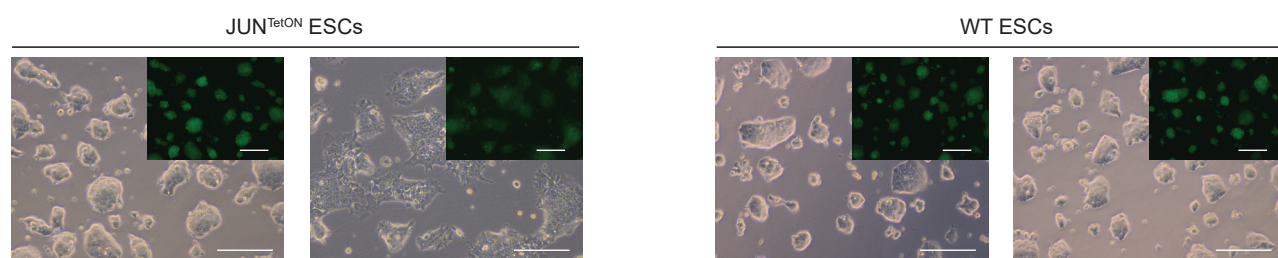

C

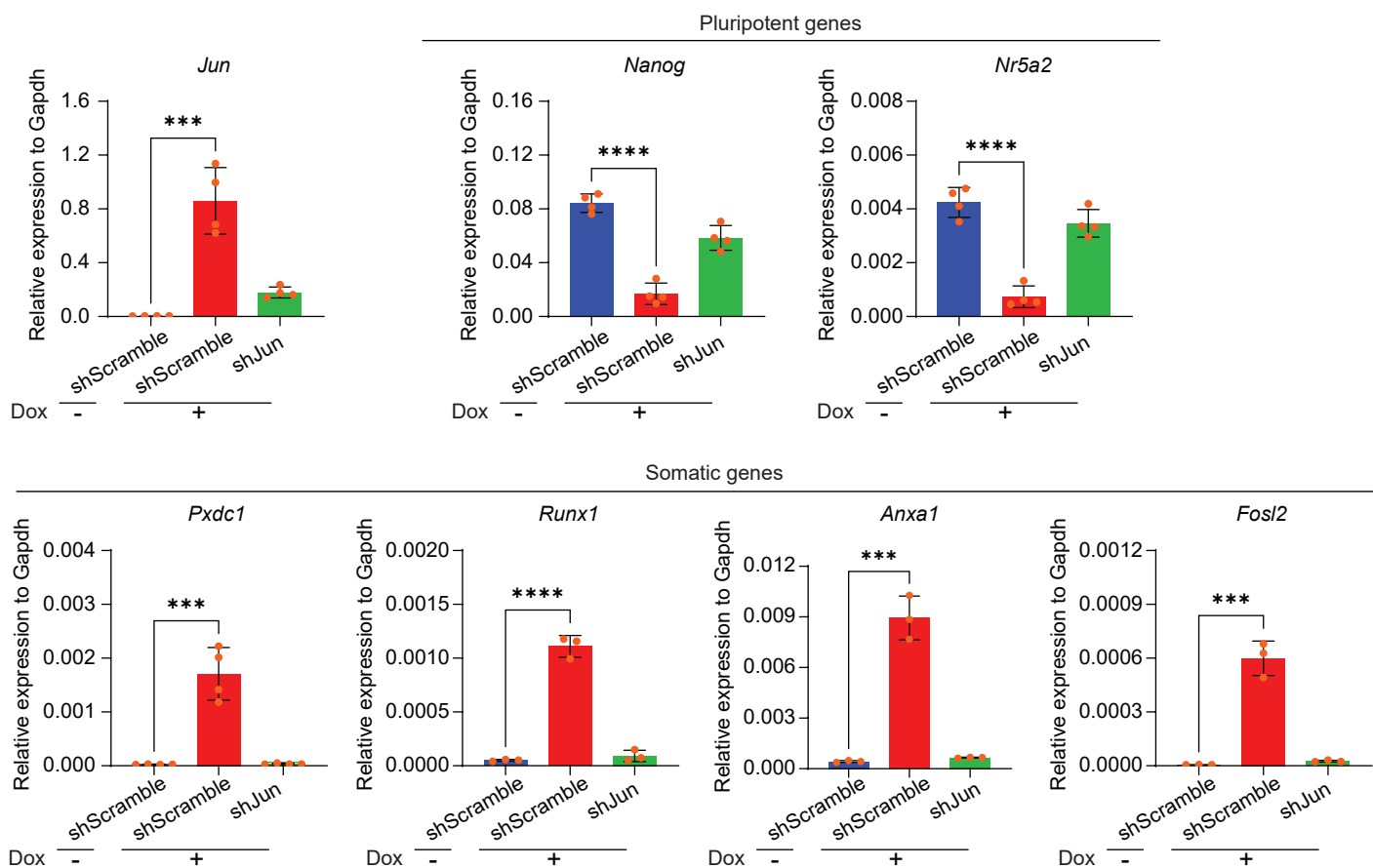

Supplement: Supplementary file 1 — Additional file 1. System specificity of JUNTetON ESCs. [file 13578_2022_827_MOESM1_ESM.pdf]

Fig. S2

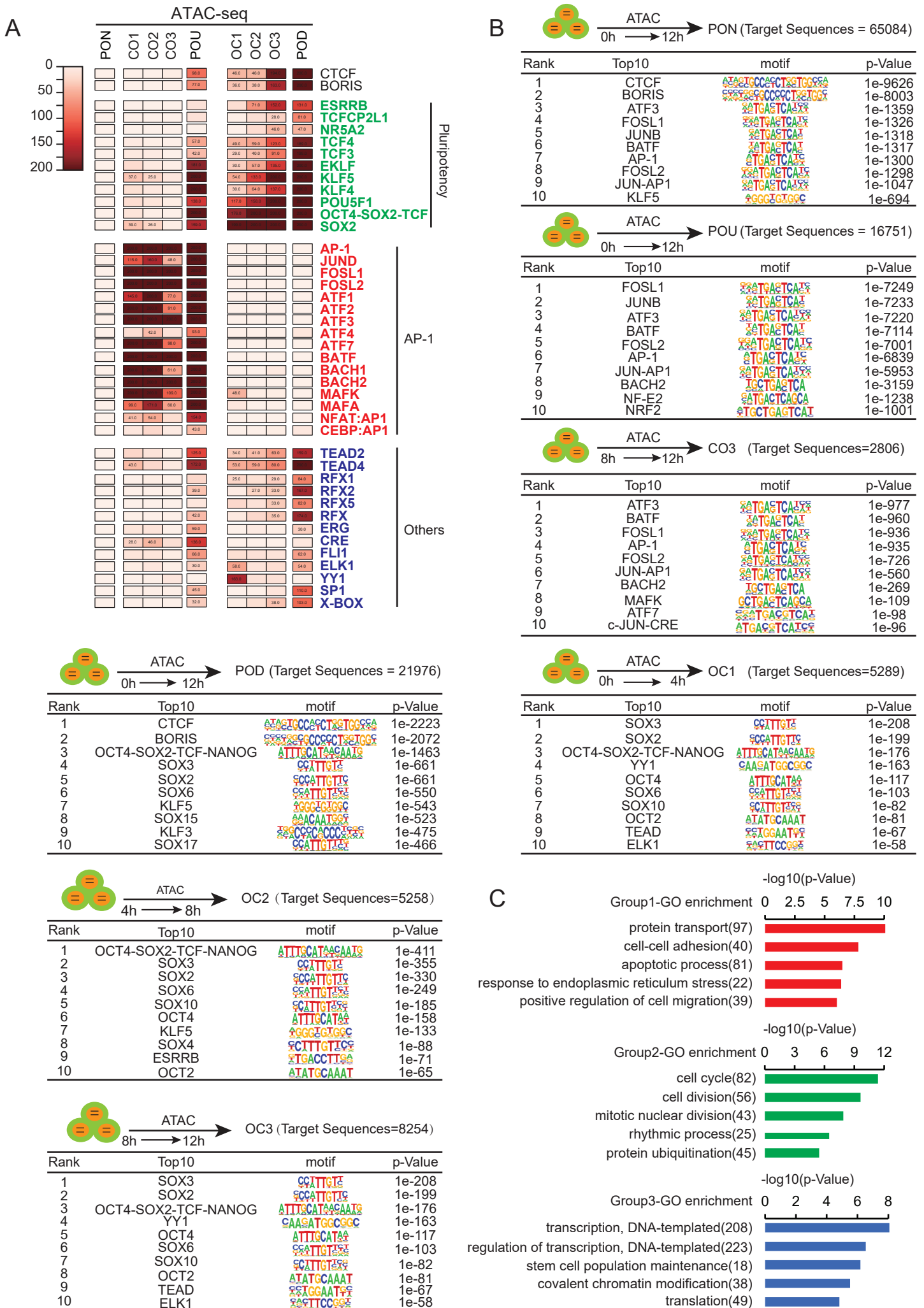

Supplement: Supplementary file 2 — Additional file 2. ATAC motif analysis and RNA-seq Gene ontology. [file 13578_2022_827_MOESM2_ESM.pdf]

Fig. S4

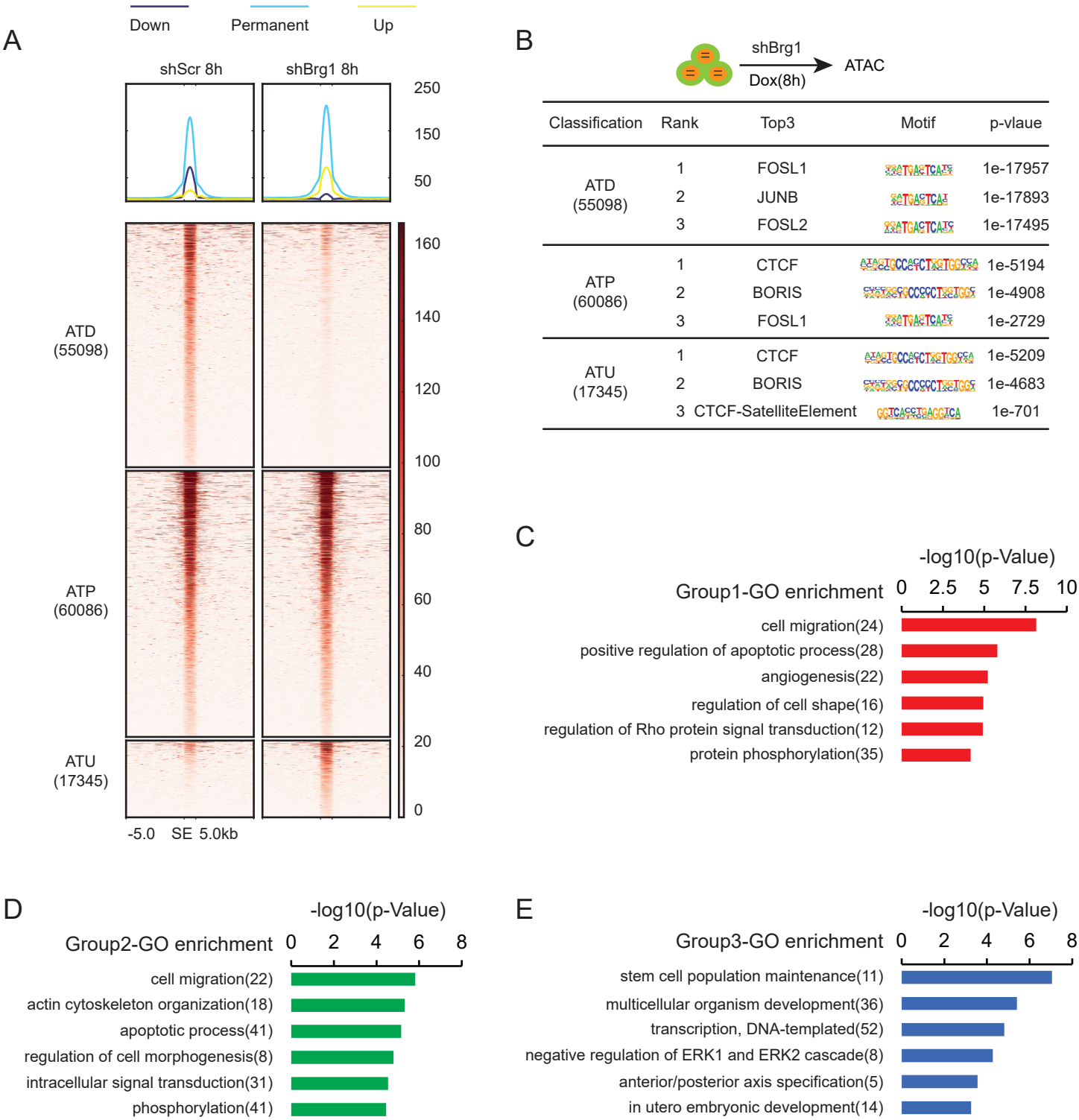

Supplement: Supplementary file 4 — Additional file 4. BRG1 is required for JUN to open chromatin. [file 13578_2022_827_MOESM4_ESM.pdf]

Fig. S5

A

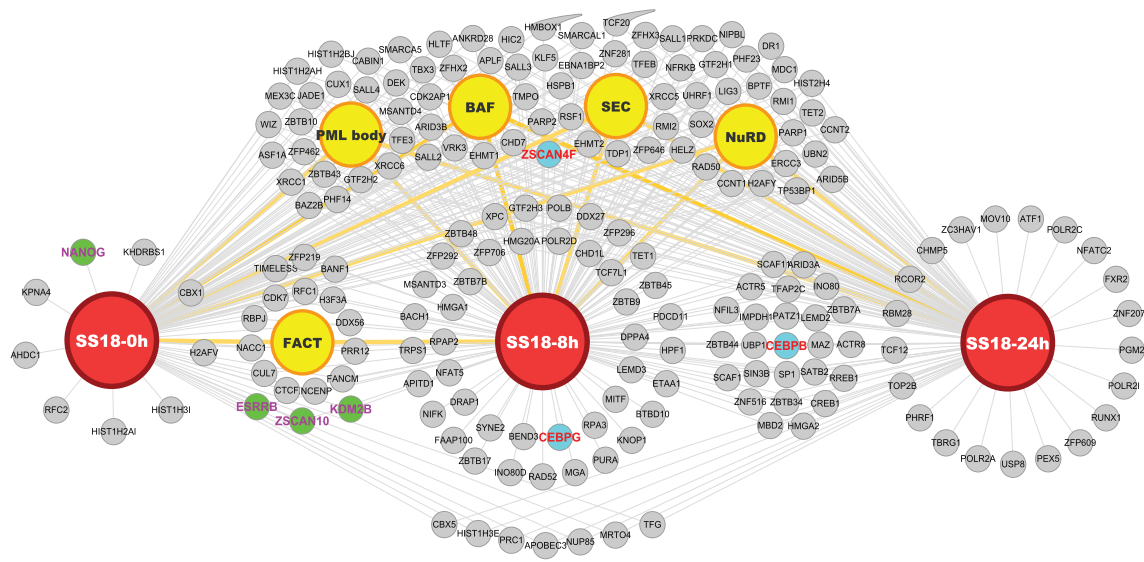

B

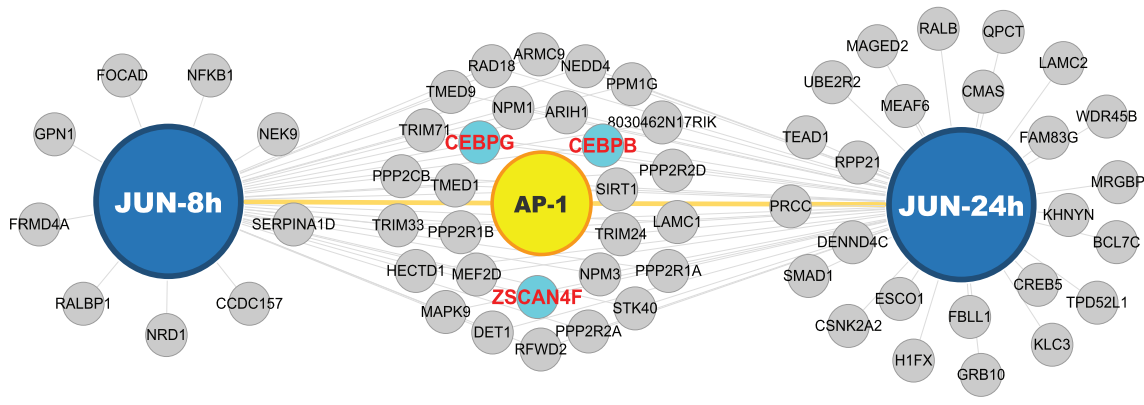

C

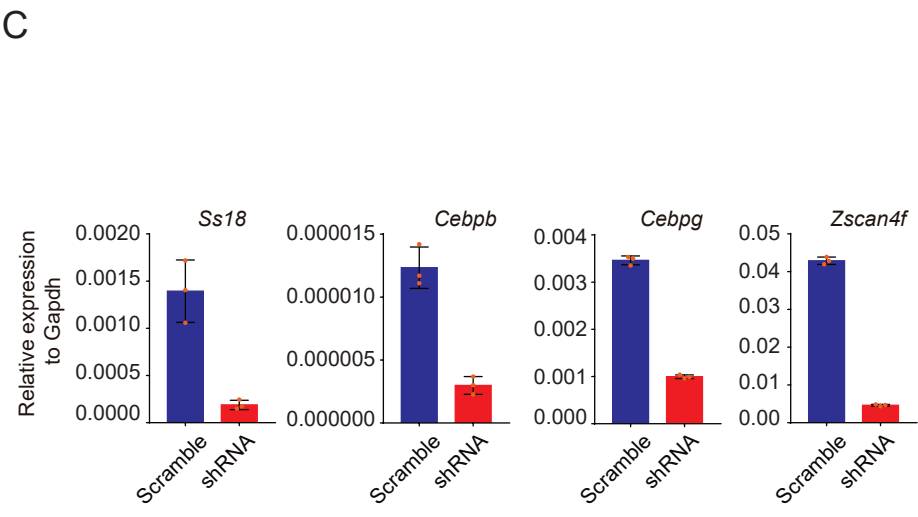

D

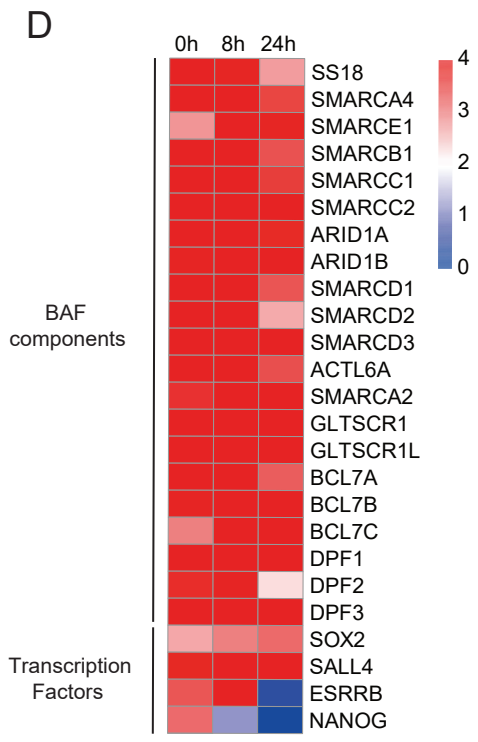

Supplement: Supplementary file 5 — Additional file 5. SS18/BAFs and JUN form independent complexes. [file 13578_2022_827_MOESM5_ESM.pdf]

Fig. S6

A

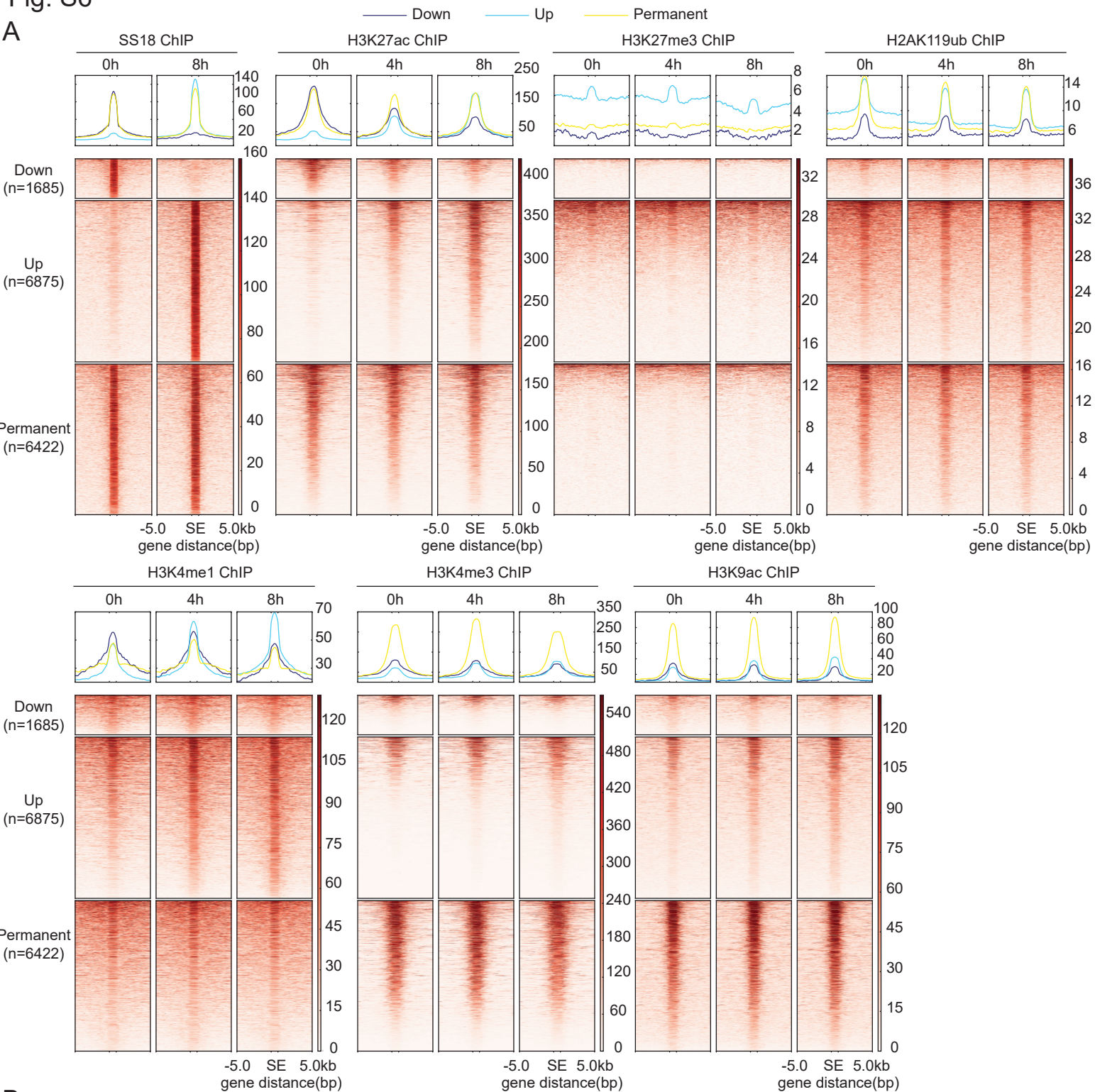

B

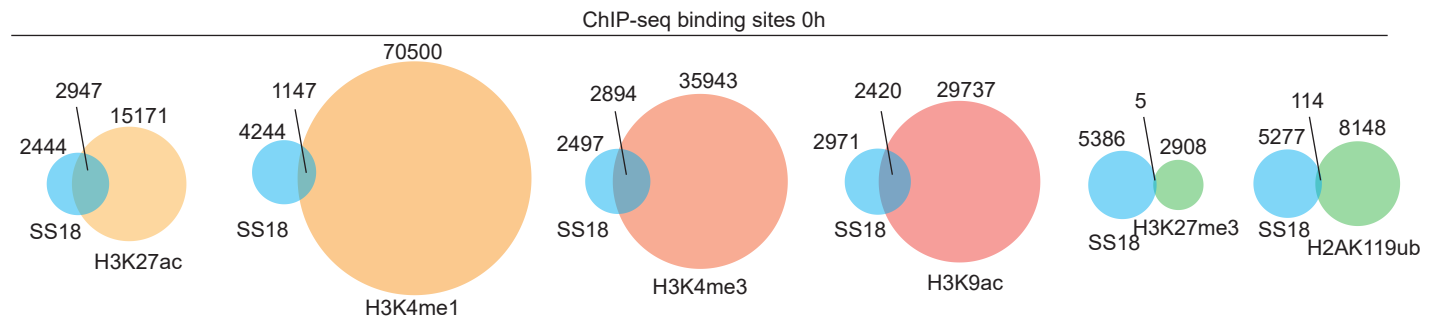

C

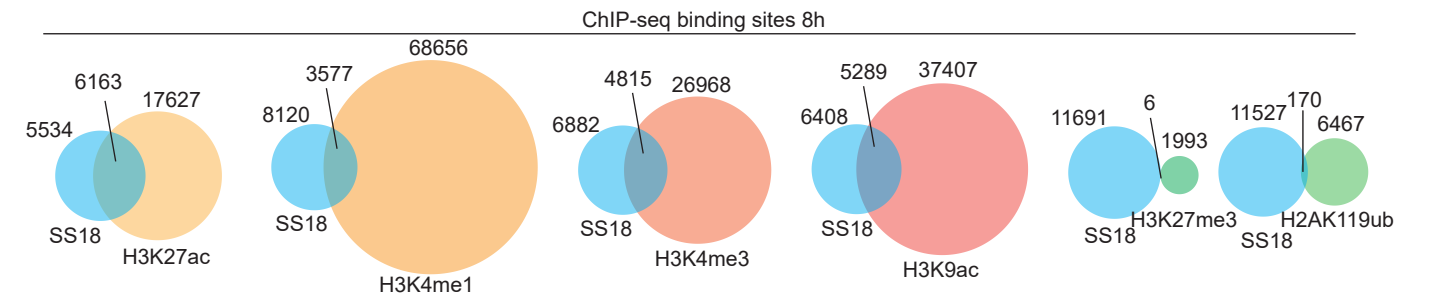

Supplement: Supplementary file 6 — Additional file 6. H3K27ac co-occupancy with SS18 during JUN induced PST. [file 13578_2022_827_MOESM6_ESM.pdf]

Fig. S7

A

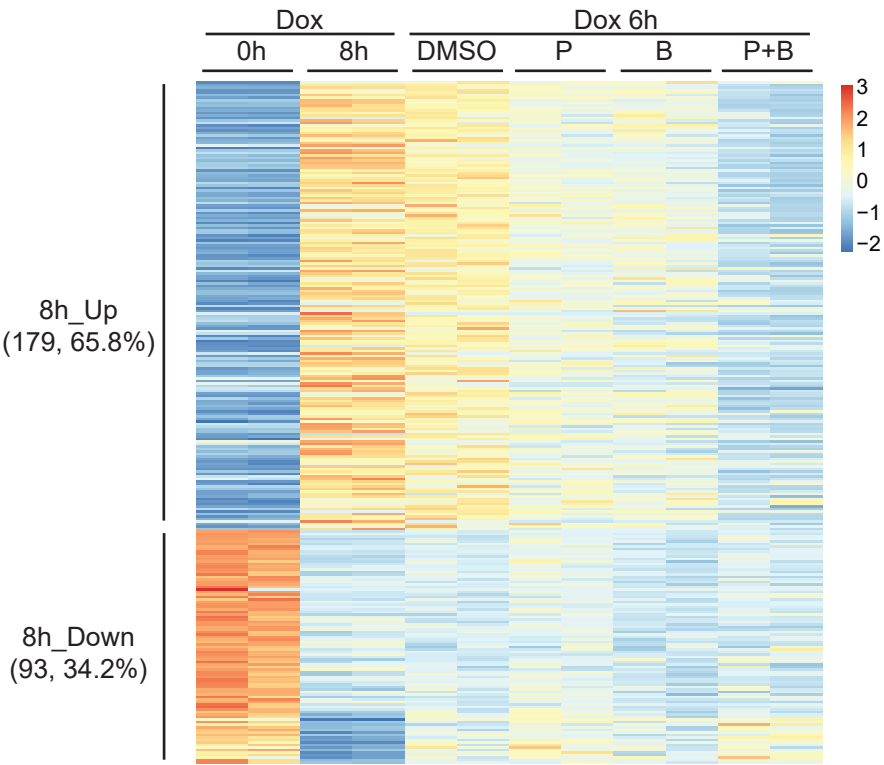

B

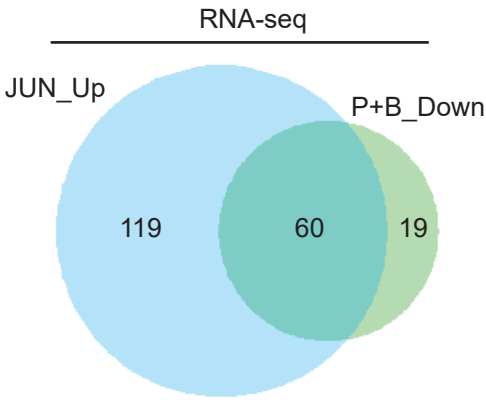

C

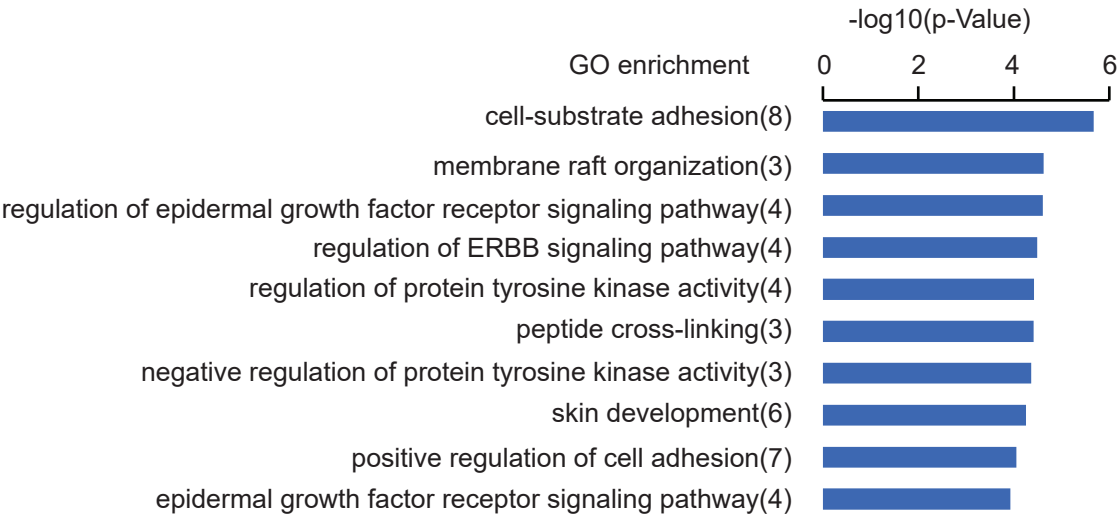

Supplement: Supplementary file 7 — Additional file 7. The BAFs’ bromodomain inhibitors impede PST. [file 13578_2022_827_MOESM7_ESM.pdf]
