# Supplementary material for: H3K27ac mediated SS18/BAFs relocation regulates JUN induced pluripotent-somatic transition
Source: Cell Biosci. 2022 Jun 16;12:89. doi: 10.1186/s13578-022-00827-1 (PMC9204951; doi:10.1186/s13578-022-00827-1)
Supplement: Supplementary file 3 — Additional file 3. JUN motif analysis. [file 13578_2022_827_MOESM3_ESM.pdf]

Fig. S3

A

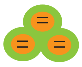

Dox(8h) → JUN ChIP-seq (peaks=6154)

Rank	Top10	motif	p-Value
1	FOSL1	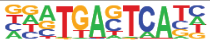	1e-3593
2	FOSL2	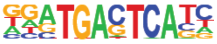	1e-3550
3	JUNB	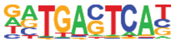	1e-3532
4	ATF3	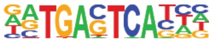	1e-3479
5	BATF	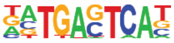	1e-3421
6	AP-1	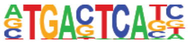	1e-3272
7	JUN-AP1	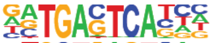	1e-3200
8	BACH2	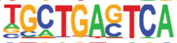	1e-1696
9	MAFK	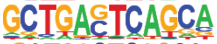	1e-601
10	NF-E2	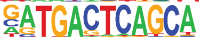	1e-545
