# Supplementary material for: H3K27ac mediated SS18/BAFs relocation regulates JUN induced pluripotent-somatic transition
Source: Cell Biosci. 2022 Jun 16;12:89. doi: 10.1186/s13578-022-00827-1 (PMC9204951; doi:10.1186/s13578-022-00827-1)
Supplement: Supplementary file 14 — Additional file 14. Descriptions text (Please replace this with a new additional file 14 in the attachments file which we made a minor correction). [file 13578_2022_827_MOESM14_ESM.docx]

**Additional file 1: Figure. S1 System specificity of JUN^TetON^ ESCs**

1. Pluripotent genes and somatic genes expression change in WT OG2 ESCs and JUN^TetON^ ESCs with the 8 hours treatment of 2μg/ml Dox. Data are mean ± s.d., two-sided, unpaired t test; n = 3 independent experiments. **p < 0.01, ****p < 0.0001.
2. representative images of OG2 and JUN^TetON^ ESCs cell morphology were obtained after Dox treatment for 8hrs and Dox was removed and cultured for 48hrs. Three biological copies. Scale bar, 120μm.
3. Knockdown efficiency of *Jun* shRNA and the effect of *Jun* knockdown on pluripotency genes and somatic genes. Data are mean ± s.d., two-sided, unpaired t test; n = 3 independent experiments. **p < 0.01, ****p < 0.0001.

**Additional file 2: Figure. S2 ATAC motif analysis and RNA-seq Gene ontology.**

1. Motif of time course ATAC-seq. CO1-3 are enriched with AP-1 related motif, OC1-3 are enriched with Pluripotency related motif.
2. Motif analysis for PON, POU, POD, OC1, OC2, OC3, CO3 during Jun based PST. Table list for the top10 most enrichment motifs.
3. GO analysis for Group1-3 of Fig. 1E, Gene number each GO term number show in bracket.

**Additional file 3: Figure. S3 JUN motif analysis.**

1. ChIP-seq analysis for Jun at 8h during Jun based PST. Table list for the top10 most enrichment motif. Peaks number=6154.

**Additional file 4: Figure. S4 BRG1 is required for JUN to open chromatin.**

- 1. Heatmap of ATAC-seq during Jun plus/minus Brg1 knock down Jun based PST 8h. Based on chromatin accessibility dynamic changes between shScramble and shBrg1, peaks divided into 3 groups, Down, Permanent and up, FC2.
  2. Table list for the top3 most enrichment motif of each group.
  3. GO analysis for group1 of Fig. 3B. Genes of each GO term number show in bracket.
  4. GO analysis for group2 of Fig. 3B. Genes of each GO term number show in bracket.
  5. GO analysis for group3 of Fig. 3B. Genes of each GO term number show in bracket.

**Additional file 5: Figure. S5 SS18/BAFs and JUN form independent complexes**

1. The network represents the SS18-Centric Protein Complex at different time points during Jun based PST. Complexes are shown as yellow circles. Some representative dynamic ones of transcription factors at 0hrs and 8hrs were highlighted by green. BAF, BAF complex; SEC, super elongation complex; FACT, FACT complex. The three shared proteins at 8hrs during PST within SS18-Centric Protein Complex and JUN-Centric Protein Complex were marked by cyan.
2. The network represents the JUN-Centric Protein Complex at different time points during Jun based PST. AP-1 family is shown as yellow circles. The three shared proteins at 8hrs during PST within SS18-Centric Protein Complex and JUN-Centric Protein Complex were marked by cyan.
3. The knockdown efficiency were detected by real-time quantification PCR at 8hrs during PST upon the knockdown of the shared three proteins. Data are mean±s.d., two tailed, unpaired t-test; n=3 independent experiments, *** P<0.001.
4. Heatmap showed the dynamics interaction between all components in BAF complex and SS18 during PST.

**Additional file 6: Figure. S6 H3K27ac co-occupancy with SS18 during JUN induced PST**

1. The combination analysis for the change of SS18, H3K27ac, H3K4me1, H3K4me3, H3K27me3, H3K9ac, H2AK119ub ChIP-seq during JUN induced PST. SE stands for TSS and TES.
2. Veen plot shows occupancy between SS18 and histone modification in ESCs.
3. Veen plot shows occupancy between SS18 and histone modification after JUN induced 8h.

**Additional file 7: Figure. S7 The BAFs’ bromodomain inhibitors impede PST.**

1. Heatmap of RNA-seq of Jun induced 8h and treated 6h with PFI-3(P), BI-9564(B) and P+B(PB). DEG were divided according dox+8h verus Dox+0h, fold change 1.5, p adjust-value 0.01.
2. Veen plot show overlap genes between upregulated in Jun induced and downregulated in PB treatment. DEG in JUN: Fold change 1.5, p adjust-value 0.01, DEG in PB: Fold change 1.5, p adjust-value 0.05.
3. Plot of top 10 GO BP enrichment for 60 overlap genes of Figure. S5B.

**Additional file 8: Table. S1 shRNA target sequences used in this study.**

**Additional file 9: Table. S2 RT-qPCR primers used in this study.**

**Additional file 10: Table. S3 IP-MS data in this study.**

**Additional file 11: Table. S4 RNA-seq data in this study.**

**Additional file 12: Table. S5 ATAC-seq TSS binding sites data in this study.**

**Additional file 13: Table. S6 Antibody used in this study.**
